# Estimating a Physiological Lung Function Score and Biological Sex Using Pulmonary Function Tests and Machine Learning: Retrospective Study

**DOI:** 10.2196/89060

**Published:** 2026-06-01

**Authors:** Patrick W Johnson, Zachary S Quicksall, Jieun Lee, Augustine S Lee, Kaiser G Lim, Victor E Ortega, Shivaram Poigai Arunachalam, Scott A Helgeson

**Affiliations:** 1 Department of Quantitative Health Sciences, Mayo Clinic Hospital, 4500 San Pablo Road S, Jacksonville, FL, 32224, United States, 1 9049536728; 2 Department of Medicine, Division of Pulmonary Medicine, Mayo Clinic Hospital, Rochester, MN, United States; 3 Department of Medicine, Division of Pulmonary Medicine, Mayo Clinic Hospital, Phoenix, AZ, United States

**Keywords:** artificial intelligence, machine learning, pulmonary function test, age, gender, spirometry

## Abstract

**Background:**

Sex and age have long been known to affect lung function. Several biological variables and anatomical factors may contribute to sex- and age-related differences in pulmonary metrics.

**Objective:**

We hypothesized that a machine learning model could be trained to predict a person’s lung age and self-reported sex using pulmonary function test data.

**Methods:**

We retrospectively analyzed complete pulmonary function tests from 6392 healthy adults across 3 Mayo Clinic regions. Four models of increasing complexity were trained using gradient-boosted machines to predict chronological age and biological sex. Model interpretability was assessed using Shapley additive explanation values and partial dependence plots. Quantile regression was used to estimate reference percentiles for predicted lung age.

**Results:**

The best-performing age model (model 4, inclusive of time-series features) achieved a root mean square error of 7.01 years (95% CI 6.73‐7.30) and a mean absolute error of 5.55 years (95% CI 5.32‐5.80). The best-performing sex classification model (model 4) achieved an area under the curve of 0.981 (95% CI 0.975‐0.988), sensitivity of 91.7% (95% CI 89.0%‐93.9%), and specificity of 95.6% (95% CI 93.9%‐97%). Key predictors for lung age included residual volume as a percentage of total lung capacity (TLC), forced expiratory volume in 1 second, and alveolar volume. For sex classification, peak expiratory flow, height, and age were among the most influential features. Age-stratified evaluation showed the overestimation of lung age in younger adults and underestimation in older adults. Predicted lung age increased broadly with chronological age, and quantile regression provided normative reference ranges.

**Conclusions:**

Applying artificial intelligence to pulmonary function data allows the prediction of a patient’s sex and estimation of lung age. The ability of an artificial intelligence algorithm to determine physiological lung age, with further validation, may serve as a measure of overall respiratory health.

## Introduction

Pulmonary function tests (PFTs) are a series of noninvasive procedures that assess the overall function and health of a patient’s lungs. A complete PFT can measure many variables reflecting different aspects of the patient’s ventilatory and gas exchange function. Deviation from population-based normative values is fundamental to the diagnosis, management, and prognosis of a variety of respiratory disorders. Several physiologic and pathologic patterns are generated by combinations of lung function variables from PFTs. In response to this latter concern, the concept of “lung age” derived from PFTs was developed as a means of “summarizing” the patient’s lung function and making it more intuitive to patients [1-7]. Because it is derived from multiple measures from the PFT, lung age may be more reflective of the patient’s overall pulmonary physiology. Scientifically, lung age has shown clinical importance in a randomized controlled trial by increasing smoking cessation [8]. The current methodology to calculate lung age depends on multiple linear regression methods and uses discrete variables obtained from the spirometry and sometimes additional clinical parameters [3,4].

Lung age is a single summary variable useful in patient education, research, and clinical practice [9-13]. For example, in chronic obstructive pulmonary disease (COPD), the forced expiratory volume in 1 second (FEV1) assesses the severity of obstruction, while in idiopathic pulmonary fibrosis (IPF), the forced vital capacity (FVC) reflects lung capacity and guides prognosis and management [10-13]. Although FEV1 and FVC are critical variables in obstructive or restrictive disorders, other variables, such as the residual volume (RV) or the diffusing capacity, can provide additional information on the patient’s respiratory function. It is also not uncommon to have mixed disorders present in the same patient, for example, combined pulmonary fibrosis with emphysema and IPF complicated by pulmonary arterial hypertension [14,15]. While convenient, the use of individual variables may therefore not communicate the breadth of the patient’s lung pathology, and importantly, these variables are conceptually less meaningful to patients who often lack the requisite medical literacy to understand respiratory pathophysiology.

PFTs reveal notable differences between biological sexes due to anatomical and physiological variations, impacting respiratory health. Men typically exhibit larger lung volumes and higher absolute values for FVC and FEV1 even when adjusted for age and height, while women often have smaller airway diameters and reduced expiratory flow rates [16-18]. A population study analyzed 3250 patients and found that sex differences in breathlessness are explained by absolute FEV1 values [16]. Given these differences, machine learning techniques are well-suited to predict patient sex based on pulmonary function test data.

In this investigation, we aimed to create an accurate lung age and sex prediction tool by incorporating all key physiological measures from the complete PFT and applying machine learning methods. We report here the development and validation of a machine learning–assisted lung age and sex prediction tool.

## Methods

### Study Population and Data Sources

This retrospective study evaluated all adult patients (aged 18 years or older) who underwent at least 1 PFT between July 2001 and May 2024 at Mayo Clinic in Florida, Rochester, and northwest Wisconsin.

### Ethical Considerations

The study was deemed exempt by the Mayo clinic institutional review board (IRB #24‐001843), with a waiver of consent. Individuals under 18 years of age and patients who opted out of authorizing their data for research use were excluded from the analysis. Demographic information, diagnostic codes, procedure records, and flowsheet data were electronically extracted from the electronic health record system. PFT data were obtained using SentrySuite equipment (Vyaire Medical) and securely stored in an on-premises research database. All data were deidentified for this study, and no compensation was provided to the participants.

### Derivation of the Healthy Training Cohort

To ensure that the models learned patterns representative of healthy pulmonary physiology, we derived a training cohort composed of patients without clinical or physiological evidence of respiratory disease. This allowed lung age and sex predictions to reflect normal variation rather than pathological changes. PFTs and associated patient records were evaluated according to the following inclusion and exclusion criteria.

Only baseline, complete PFTs, including spirometry, lung volume, and diffusion capacity assessments, were included. For patients with multiple complete PFTs in the medical record, only the earliest test was selected. When multiple curves were recorded during a single session, only the best curve was retained and used for this study. Tests were excluded if they did not meet the criteria for normal lung function as defined by the American Thoracic Society guidelines or if they were classified under GOLD criteria as category 1 [10,19]. Tests were also excluded if they were missing key metrics (eg, FEV₁, FVC, or FEV₁/FVC) or if measured values fell outside the predicted lower and upper limits of normal.

Patients were excluded if they had any documented history of chronic obstructive pulmonary disease, asthma, interstitial lung disease, sarcoidosis, bronchiectasis, cystic fibrosis, pleural effusions, malignant neoplasms involving the lung, bronchus, or trachea, or any other documented lung disease. Those with a history of tobacco use or prior lung transplant were also excluded. PFTs associated with a Modified Medical Research Council Dyspnea Scale score >1 at the time of testing were excluded. Finally, patients were excluded if their BMI at the time of testing was classified as underweight or as class 2 or 3 obesity, or if their recorded height fell outside the range of 1.21 to 2.13 m [19].

### Model Data and Development

Before model development, the dataset was randomly split into mutually exclusive training (n=5134, 80%) and testing (n=1258, 20%) sets, ensuring that each patient appeared in only 1 set. All modeling and hyperparameter tuning were performed using the training set, while final performance was assessed on the independent testing set. Baseline demographic and physiologic characteristics were compared between the training and testing sets using standardized mean differences, with a threshold of <0.1 indicating acceptable balance [20].

For each outcome, chronological age (regression) and sex (classification), four progressively complex models were constructed:

1. Model 1 (basic spirometry): Included only demographic variables and features routinely available in portable and clinical spirometry
2. Model 2 (expanded spirometry): Incorporated additional spirometry-derived variables beyond those included in model 1
3. Model 3 (full discrete PFT): Included all variables from spirometry, lung volume, and diffusion capacity tests
4. Model 4 (time series): Included all variables from model 3, along with time-series features derived from expiratory flow-volume curves (eg, autocorrelation, spikiness, flatness, Hurst exponent, and nonlinearity)

A complete list of input features used in each model is provided in Table S1 in Multimedia Appendix 1.

Each of the 8 models (4 predicting age and 4 predicting sex) was trained using gradient-boosted machines. Hyperparameters, including learning rate, maximum tree depth, sample rate, column sample rate, and the number of trees, were optimized using a grid search of 144 candidate combinations with 5-fold cross-validation. For age prediction, the model with the lowest average root mean square error (RMSE) across folds was selected. For sex classification, the model with the highest average area under the curve (AUC) was selected. The final tuned hyperparameters for each model are shown in Table S9 in Multimedia Appendix 1.

Final model performance was assessed using the independent test set. For age prediction, performance was summarized using RMSE, mean absolute error (MAE), and the coefficient of determination (*R*²). Predicted values were compared with chronological age using scatter plots and heat maps to visualize accuracy across the age spectrum. Pairwise differences in absolute prediction errors were assessed using the Wilcoxon signed-rank test on the hold-out test set. Performance was also benchmarked against the existing lung age model published by Parkes et al [8]. For sex classification, receiver operating characteristic (ROC) curves were generated, and AUC was calculated alongside standard binary classification metrics. Pairwise comparisons of AUC were conducted using DeLong’s paired test for correlated ROC curves.

To evaluate whether model accuracy varied across the age spectrum, we computed age-stratified performance on the independent test set for models 3 and 4. Patients were grouped into 7 age bins (18‐29, 30‐39, 40‐49, 50‐59, 60‐69, 70‐79, and 80 years or older). Within each bin, we calculated sample size, RMSE, MAE, mean signed prediction error, and median signed prediction error, defining signed error as predicted age minus chronological age. Bin-level 95% CIs were estimated using nonparametric bootstraps with 2000 resamples.

To quantify the contribution of anthropometric variables to model performance, we performed a sensitivity analysis in which height and weight were removed from the feature sets for models 3 and 4. Separate age and sex models were retrained using the same training set, hyperparameter grid, 5-fold cross-validation strategy, and model-selection criteria as in the primary analysis. Performance was then evaluated on the unchanged independent test set and compared with the corresponding primary models.

Calibration of the sex classification models was assessed on the independent test set using reliability diagrams, the Brier score, the expected calibration error, and the Spiegelhalter *z* statistic.

To facilitate the interpretation of model predictions, Shapley additive explanation (SHAP) values were computed to quantify the contribution of each input feature. SHAP summary plots illustrated both the direction and magnitude of variable effects. Partial dependence plots were also generated for high-impact features to visualize their marginal effect on predicted outcomes, independent of other variables.

To define normative ranges for predicted lung age across the lifespan, quantile regression was applied to the full dataset (training and testing combined). Analyses were performed for 2 models—the full discrete-feature model (model 3) and the simplified spirometry model (model 1)—to provide reference curves for both the higher-performing discrete PFT model and the simpler spirometry-based model that may be more feasible in broader clinical settings. Predicted lung age was regressed on chronological age using second-degree polynomial terms, with the Barrodale and Roberts algorithm used to estimate the 10th, 25th, 50th, 75th, and 90th percentiles [21]. This approach yielded smooth, continuous percentile curves across the age spectrum.

### Software and Statistical Analysis

All data processing, statistical analyses, and model development were performed using R version 4.2.2 (R Core Team) and the H_2_O machine-learning framework (version 3.44.0.3). Reported *P* values are 2-sided, and statistical significance was defined as *P*<.05. Model performance was summarized with 95% CIs on the independent test set. For age prediction, CIs for RMSE, MAE, and *R*² were estimated using 2000 patient-level bootstrap resamples. For sex classification, AUC CIs were estimated using the DeLong method, and CIs for sensitivity, specificity, positive predictive value, and negative predictive value were estimated using exact binomial methods at a probability threshold of 0.5. Categorical predictors were retained as categorical fields, continuous predictors were not centered or scaled, and no imputation was performed. Missing predictor values were handled during gradient-boosted machine fitting.

## Results

### Study Cohort

A total of 68,707 patients underwent a complete PFT during the study period, of whom 6392 met the inclusion criteria for the healthy cohort. Key patient characteristics and PFT measurements, stratified by data split (training: n=5134; testing: n=1258), are shown in Table 1. The median age was 59.0 (IQR 46.9‐68.6; range 18.0‐95.5) years, and patients were predominantly female (n=3698, 57.9%) and Caucasian (n=5721, 89.5%). No meaningful differences were observed between the training and testing sets, with all standardized differences <0.1.

**Table 1.** Patient demographics.

Characteristics	Test (n=1258)	Train (n=5134)	Total (n=6392)	Standard difference
Age (y), median (IQR; range)	58.6 (46.8-68.1; 18.0‐94.2)	59.0 (46.9-68.7; 18.0‐95.5)	59.0 (46.9-68.6; 18.0‐95.5)	0.015
Sex, n (%)				0.004
Female	730 (58)	2968 (57.8)	3698 (57.9)	
Male	528 (42)	2166 (42.2)	2694 (42.1)	
Weight (kg), median (IQR; range)	77.2 (66.2-89.2; 43.8‐144.4)	77.5 (66.3-88.9; 43.3‐128.0)	77.4 (66.3-88.9; 43.3‐144.4)	0.018
Height (m), median (IQR; range)	1.7 (1.6-1.8; 1.5‐2.1)	1.7 (1.6-1.8; 1.4‐2.0)	1.7 (1.6-1.8; 1.4‐2.1)	0.001
Slow vital capacity, median (IQR; range)	3.6 (3.0-4.4; 1.7‐7.1)	3.6 (3.0-4.4; 1.6‐7.4)	3.6 (3.0-4.4; 1.6‐7.4)	0.006
Race, n (%)				0.062
Asian	18 (1.4)	89 (1.7)	107 (1.7)	
Black	100 (7.9)	352 (6.9)	452 (7.1)	
Caucasian	1123 (89.3)	4598 (89.6)	5721 (89.5)	
Other/mixed	12 (1)	64 (1.2)	76 (1.2)	
US Asian	5 (0.4)	31 (0.6)	36 (0.6)	
Forced vital capacity, median (IQR; range)	3.6 (3.0, 4.3; 1.6‐7.1)	3.6 (3.0, 4.4; 1.6‐7.4)	3.6 (3.0, 4.4; 1.6‐7.4)	0.004
Forced expiratory volume (1 s), median (IQR; range)	2.8 (2.4-3.4; 1.3‐5.6)	2.8 (2.3-3.5; 1.2‐6.2)	2.8 (2.3-3.5; 1.2‐6.2)	0.004
FEV1^a^/FVC^b^ ratio, median (IQR; range)	0.8 (0.8-0.8; 0.6‐1.0)	0.8 (0.7-0.8; 0.6‐1.0)	0.8 (0.7-0.8; 0.6‐1.0)	0.020
Peak expiratory flow, median (IQR; range)	7.8 (6.5-9.5; 3.0‐15.5)	7.6 (6.3-9.5; 2.4‐17.3)	7.7 (6.4-9.5; 2.4‐17.3)	0.023
Forced expiratory time, median (IQR; range)	7.8 (6.7-9.4; 1.7‐21.6)	7.8 (6.7-9.3; 1.3‐25.6)	7.8 (6.7-9.4; 1.3‐25.6)	0.005
Functional residual capacity, median (IQR; range)	3.0 (2.6-3.6; 1.5‐6.8)	3.0 (2.5-3.6; 1.0‐7.6)	3.0 (2.6-3.6; 1.0‐7.6)	0.027
Residual volume, median (IQR; range)	1.9 (1.6-2.3; 0.3‐4.6)	1.9 (1.6-2.3; 0.5‐5.4)	1.9 (1.6-2.3; 0.3‐5.4)	0.025
Diffusing capacity, median (IQR; range)	6.6 (5.6-8.1; 2.7‐14.0)	6.7 (5.5-8.2; 2.0‐16.7)	6.7 (5.5-8.2; 2.0‐16.7)	0.012
Alveolar volume, median (IQR; range)	4.9 (4.2-5.8; 2.5‐9.5)	4.9 (4.2-5.9; 2.4‐9.6)	4.9 (4.2-5.8; 2.4‐9.6)	0.005
Corrected diffusing capacity				0.007
Missing, n	312	1280	1592	
Median (IQR; range)	6.8 (5.7-8.2; 2.7‐13.7)	6.8 (5.6-8.3; 2.1‐15.6)	6.8 (5.6-8.3; 2.1‐15.6)	

a FEV1: forced expiratory volume in 1 second.

b FVC: forced vital capacity.

### Age Prediction Models

Among the 4 age prediction models, model 4 (time-series features) achieved the highest accuracy, with an RMSE of 7.01 years (95% CI 6.73‐7.30) and an MAE of 5.55 years (95% CI 5.32‐5.80). Model 3 (discrete PFT variables) followed closely, with an RMSE of 7.19 years (95% CI 6.90‐7.49) and an MAE of 5.69 years (95% CI 5.45‐5.95). Models 1 and 2, based on basic and expanded spirometry, respectively, demonstrated higher errors, with RMSEs of 9.29 years (95% CI 8.87‐9.68) and 9.13 years (95% CI 8.74‐9.49), and MAEs of 7.32 years (95% CI 6.99‐7.65) and 7.27 years (95% CI 6.96‐7.58), respectively (Table S2 in Multimedia Appendix 1).

**Figure 1.**
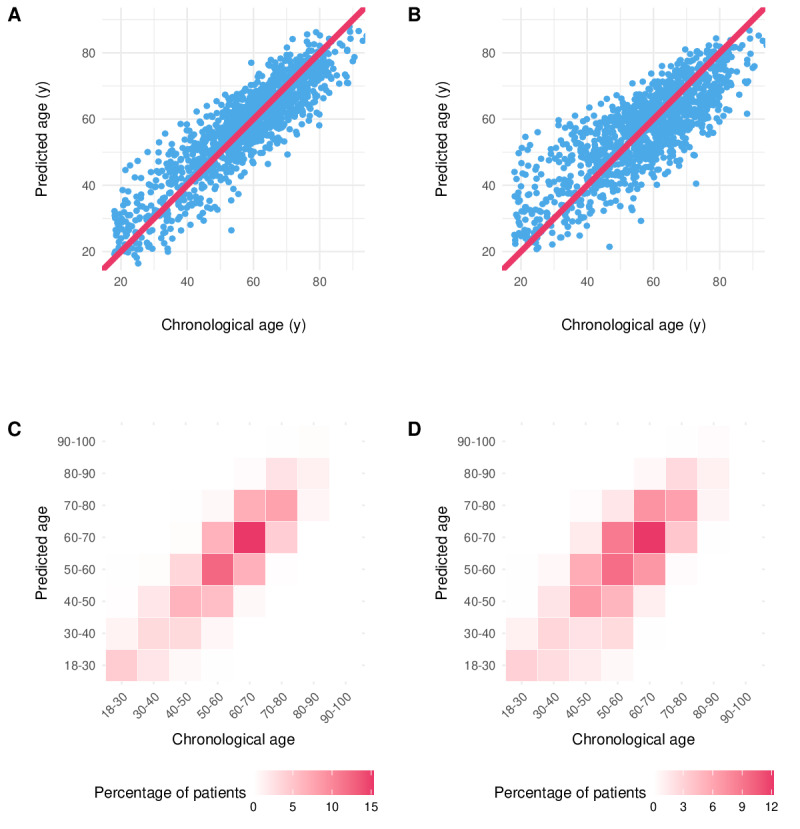
Predicted versus chronological age across models. (A and B) Scatter plots of predicted versus actual age for 2 representative models, with the line of identity (red) shown for reference. (C and D) Binned heatmaps showing the joint distribution of predicted and actual age, with color intensity representing the proportion of patients in each bin. Left panels: basic spirometry model (model 1); right panels: discrete pulmonary function test (PFT) model (model 3). Models tended to overestimate lung age in younger adults and underestimate lung age in older adults, with the smallest errors near the cohort median age.

Pairwise comparisons of absolute errors showed that model 4 outperformed all other models (*P*<.05). Model 3 also outperformed both simpler models (*P*<.05), whereas models 1 and 2 did not differ significantly (*P*=.33). All models outperformed the prior lung age model published by Parkes et al [8] (*P*<.05). Figure 1A and B display predicted versus chronological age for models 1 and 3, respectively, and Figure 1C and D show the corresponding binned heat maps.

Age-stratified evaluation on the independent test set showed an age-dependent signed error in both models 3 and 4. In model 4, predictions were higher than chronological age in younger adults and lower than chronological age in older adults, consistent with regression to the mean toward the cohort median age. The mean signed error was 4.64 years (95% CI 3.08‐6.08) in participants aged 18 to 29 years and −6.47 years (95% CI −7.78 to −5.22) in participants aged 80 years or older. Errors were the lowest in the 60‐ to 69-year age group. Similar patterns were observed for model 3 (Tables S5 and S6 in Multimedia Appendix 1; Figures S2-5S in Multimedia Appendix 1).

**Figure 2.**
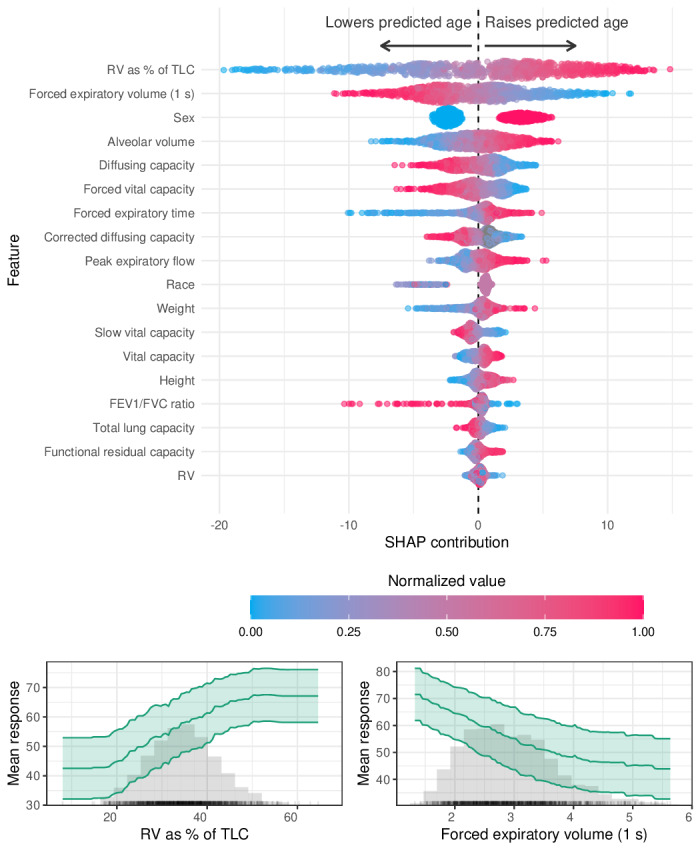
Feature contributions to predicted lung age (model 3). Top: Shapley additive explanation (SHAP) summary plot showing the relative importance and directional influence of features on predicted lung age. Each point represents an individual prediction, colored by the normalized value of the corresponding feature. Features at the top had the greatest overall impact on model output. Bottom: Partial dependence plots (PDPs) for 2 influential features—residual volume (RV) as a percentage of total lung capacity (TLC) and forced expiratory volume in 1 second (FEV1)—illustrating their marginal effects on predicted lung age. Shaded bands represent 95% CIs. FVC: forced vital capacity; TLC: total lung capacity.

In model 3, the most influential features for predicting lung age included RV as a percentage of TLC, FEV₁, and alveolar volume. Additional contributors included diffusing capacity, sex, FVC, and anthropometric variables, such as height and weight. Higher RV% and lower FEV₁ values were associated with older predicted lung age. Partial dependence plots for RV as a percentage of TLC and FEV₁ revealed nonlinear associations with the model output (Figure 2).

Anthropometric ablation analyses showed only modest changes in age-prediction performance after the removal of height and weight. For age prediction, RMSE changed from 7.19 to 7.36 years in model 3 and from 7.01 to 7.10 years in model 4, while MAE changed from 5.69 to 5.79 years in model 3 and from 5.55 to 5.59 years in model 4 (Table S7 in Multimedia Appendix 1).

**Figure 3.**
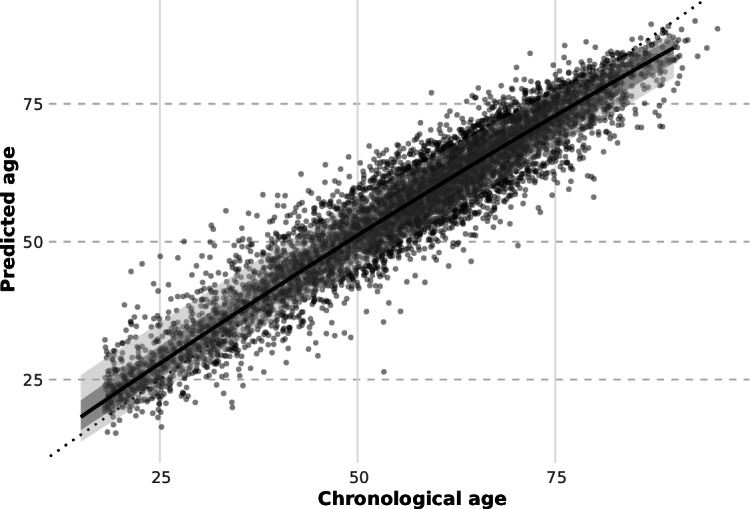
Quantile regression of predicted lung age (model 3). Predicted lung age is plotted against chronological age, with quantile regression curves representing the 10th, 25th, 50th (median), 75th, and 90th percentiles. The central solid line shows the smoothed median prediction, with shaded bands illustrating the interquartile (25th-75th) and 10th-90th percentile ranges. Percentile estimates were derived using second-degree polynomial fits on the full dataset.

Predicted lung age increased broadly with chronological age across the cohort (Figure 3). Smoothed quantile regression curves captured the distribution of predicted lung age at each age, with the 10th to 90th percentile bands centered around the median. Percentile estimates at 10-year intervals are summarized for both models 1 and 3 in Table S3 in Multimedia Appendix 1.

### Sex Prediction Models

All 4 models demonstrated high discriminatory performance in predicting biological sex. Model 1 (basic spirometry) achieved an AUC of 0.965 (95% CI 0.955‐0.975), and model 2 (expanded spirometry) improved to 0.978 (95% CI 0.970‐0.986). Models 3 (discrete PFT variables) and 4 (time-series features) performed similarly, with each achieving an AUC of 0.981 (model 3: 95% CI 0.974‐0.988; model 4: 95% CI 0.975‐0.988) (Table S4 in Multimedia Appendix 1).

Pairwise comparisons showed that model 1 performed worse than models 2, 3, and 4. Model 4 outperformed model 2 (*P*=.02), whereas the difference between models 2 and 3 was marginal (*P*=.06) and models 3 and 4 did not differ significantly (*P*=.67).

Model 4 achieved a sensitivity of 91.7% (95% CI 89.0%‐93.9%), specificity of 95.6% (95% CI 93.9%‐97.0%), positive predictive value of 93.8% (95% CI 91.4%‐95.7%), and negative predictive value of 94.1% (95% CI 92.1%‐95.7%). Figure S1 in Multimedia Appendix 1 presents the ROC curves and classification performance for models 1 and 3. Performance was consistent across racial and age subgroups, with no significant interaction effects detected (*P*>.05). Calibration was favorable overall across the sex models, with Brier scores ranging from 0.049 to 0.066 and the expected calibration error below 0.04 for all models (Table S10 in Multimedia Appendix 1; Figure S6 in Multimedia Appendix 1).

**Figure 4.**
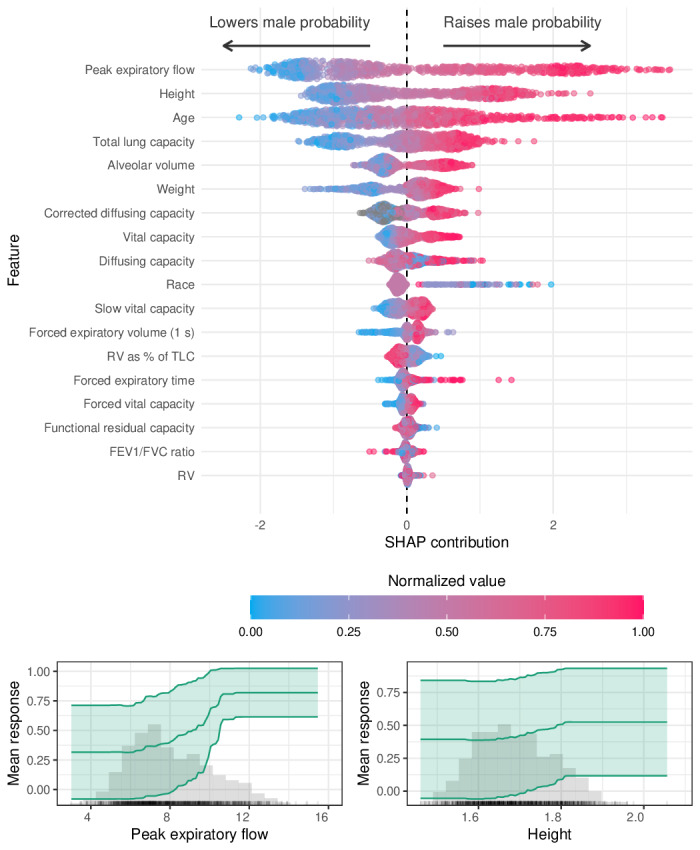
Feature contributions to sex classification (model 3). Top: Shapley additive explanation (SHAP) summary plot showing the relative importance and directional impact of individual features on the predicted probability of male sex. Each point represents a single patient, with color indicating the normalized value of the corresponding feature. Features at the top had the greatest influence on model predictions. Bottom: Partial dependence plots (PDPs) for 2 top features—peak expiratory flow and height—illustrating their marginal effects on model output. Shaded areas represent 95% CIs. FVC: forced vital capacity; TLC: total lung capacity.

The most influential features in model 3 for sex classification included peak expiratory flow, height, and age. Additional contributors included TLC, alveolar volume, weight, and corrected diffusing capacity. SHAP distributions revealed distinct patterns by predicted sex, with peak expiratory flow and height exhibiting clear directional contributions toward male versus female classification. Partial dependence plots further illustrated the marginal effects of these features on the probability of male sex (Figure 4).

After the removal of height and weight, sex classification performance remained essentially unchanged. AUC was 0.980 (95% CI 0.973‐0.986) for the no-anthropometry version of model 3 and 0.981 (95% CI 0.974‐0.987) for the no-anthropometry version of model 4, compared with 0.981 for both primary models (Table S8 in Multimedia Appendix 1).

## Discussion

Our study offers an innovative perspective on leveraging machine learning to derive a single, intuitive index—lung age—from PFT data. In doing so, we extend the concept of lung age, which has historically relied on linear regression and a limited number of spirometric variables such as FEV₁ and FVC [1-5,7,8,22,23]. Although traditional lung age estimates have informed clinical practice, particularly in motivating smoking cessation, their scope may be inadequate for detecting mixed respiratory disorders or capturing the complexity of progressive diseases, such as COPD and IPF [1,4,8,12,15,19,22,23]. By integrating additional parameters, including lung volumes and diffusing capacity, our models encapsulate broader physiologic domains, thereby providing clinicians and patients with a more complete snapshot of pulmonary function.

A distinguishing innovation in our methodology lies in the incorporation of time-series features from expiratory flow-volume curves, an approach that harnesses the temporal complexity of airflow mechanics [24]. Traditional linear models often reduce such curves to a few key values (eg, peak expiratory flow or FEV₁/FVC ratio), potentially discarding clinically relevant information hidden within continuous signals [19,25]. The use of gradient-boosted machines enabled the model to use these subtle interactions among multiple respiratory variables, resulting in significantly enhanced predictive accuracy for both age and sex classifications. Interestingly, model 3—which relied solely on tabular PFT data—offers a near-equivalent performance while being more amenable to seamless clinical adoption, such as an online calculator for diverse health care environments. In contrast, the most advanced model (model 4), which incorporated time-series data, exhibited marginally superior accuracy and may be best suited for specialized centers capable of handling more intensive data processing. These findings encourage further research to determine whether the incremental performance gains from time-series data justify the greater computational demand, especially in patient populations with established respiratory dysfunction.

Beyond its use for age estimation, these models demonstrated robust performance in sex classification, underscoring the fundamental physiological differences that exist between male and female lungs [26-28]. This high fidelity not only addresses potential gaps in demographic data encountered in large-scale epidemiological surveys but could also influence more nuanced investigations into sex-specific risks and disease trajectories [29-32]. Additionally, the extension of sex classification models to transgender populations may offer an innovative lens on how hormonal therapies or varying physiological baselines interact with lung function over time, but these questions require meticulous data collection regarding gender identity and transitions. From a clinical use perspective, precise sex classification can refine predictive models, ensuring that normative ranges and targeted interventions align more accurately with individual patient needs.

This work highlights the importance of model interpretability, which is often overlooked in the rapid adoption of machine learning approaches in health care. By leveraging SHAP and partial dependence plots, we were able to offer clinicians clear insights into how specific features (eg, RV, functional residual capacity, anthropometric variables) drive each individual prediction [33,34]. This transparency is pivotal for patient-centered communication, as it enables clinicians to contextualize changes in lung age or sex-based predictions within underlying physiologic processes and to discuss tailored management strategies. By identifying which variables exert the greatest influence on a patient’s estimated lung age, for instance, a provider might more effectively counsel on lifestyle modifications, targeted therapies, or disease-monitoring intervals. For age predictions, SHAP analysis showed that RV as a percentage of TLC, FEV₁, and alveolar volume were among the most influential features. For sex predictions, the SHAP plots showed distinct patterns in how features, such as peak expiratory flow and height, contributed to classification outcomes for male participants versus female participants. Importantly, anthropometric ablation analyses showed only modest reductions in performance after the removal of height and weight, suggesting that body size contributes to prediction but does not fully account for model performance.

Lung volumes, notably the RV and the RV/TLC ratio, increase with advancing age and may reduce the vital capacity [35]. This age-related trend aligns with the physiological principle that dynamic small airway compression caused by loss of alveolar elasticity due to aging traps gas in the lungs and elevates RV [36]. In COPD, resting pulmonary hyperinflation (RV/TLC ≥40%) correlates with older age [37]. If the increase in RV surpasses functional residual capacity, more peripheral small airways may not be ventilated, subsequently failing to participate in gas exchange and affecting the regional ventilation-perfusion ratio, which may cause the diffusion capacity of the lung for carbon monoxide to also decline with age [35]. Cross-sectional studies may overestimate or underestimate these age-related changes because of cohort and period effects, including improved measurement techniques and shifting environmental exposures. Individuals with wheezing symptoms or lower FEV₁ often exhibit higher RV/TLC, underscoring the combined role of airflow obstruction and hyperinflation [35]. Furthermore, a negative relationship between RV/TLC and diffusing capacity adjusted for lung volume (diffusion capacity of the lung for carbon monoxide/alveolar volume) points to alveolar enlargement or membrane alterations that hinder gas exchange [35]. Collectively, these findings emphasize how structural, mechanical, and biochemical factors converge to shape lung aging.

The healthy cohort was skewed toward later adulthood, and age-stratified analyses showed that model error was not uniform across the age spectrum. Both models 3 and 4 tended to overestimate lung age in younger adults and underestimate lung age in older adults, with the smallest errors near the cohort’s median age. This pattern is consistent with regression to the mean in a training distribution concentrated in middle and later adulthood. Accordingly, lung age estimates from the current model should be interpreted most cautiously at the youngest and oldest ages. Future efforts to mitigate this pattern may include age-balanced sampling during model development, expansion of healthy participants at the age extremes, post hoc recalibration of predicted lung age against chronological age, and reliability flagging for underrepresented age ranges.

The sex prediction models also demonstrated high accuracy, with model 4 achieving an AUC of 0.981, indicating robust discrimination between male and female pulmonary physiology. This performance may reflect known sex-related differences in lung size, airway diameter, and volume distribution. Key contributing features in sex prediction included the FEV1/FVC ratio, RV, and functional residual capacity, with demographic factors, such as height and weight, also influencing model predictions. Notably, peak expiratory flow and height showed distinct contributions to male and female classifications, aligning with known sex-based anatomical and physiological variations [17,18,38]. It would be interesting to apply the sex model within the transgender population.

Nonetheless, our findings have certain limitations. The retrospective study design, coupled with the inclusion of patients undergoing PFTs for clinical indications ranging from presurgical evaluation to pre–bone marrow transplant assessment to dyspnea without respiratory disease, may limit broader generalizability. In addition, because the cohort was derived from patients undergoing clinically indicated testing rather than from a population-based healthy sample, some selection bias may remain despite the rigorous healthy cohort definition. The cohort was also predominantly Caucasian, which may limit generalizability to more diverse populations. Moreover, although lung age incorporates data from multiple physiologic dimensions, real-world deployment hinges on the availability of advanced PFT equipment, consistent procedural quality, and robust data integration infrastructures, factors that might challenge adoption in resource-limited settings [39]. Ethical and data governance considerations also arise when implementing machine learning models at scale, given the sensitive nature of electronic health record systems and potential biases introduced by incomplete or inaccurate patient information [40,41].

Lung age models have the potential to change how pulmonary health is assessed, communicated, and monitored. By simplifying pulmonary function test data into a single, intuitive metric, lung age may provide a tool that enhances our understanding of disease severity, improves risk communication, and motivates behavioral change, particularly in high-risk populations such as smokers. These models may also serve as objective biomarkers for early disease detection, longitudinal monitoring, and therapeutic response, enabling clinicians to track subtle physiologic changes over time that may not be readily apparent through traditional spirometric thresholds alone. Future research could expand on these results in several ways. Prospective, longitudinal studies might elucidate how lung age evolves longitudinally over multiple PFTs, particularly in conditions marked by gradual pulmonary decline, such as IPF or advanced COPD [42]. In such contexts, lung age might serve as a surrogate end point for clinical trials, offering a more personalized measure of disease progression and treatment response than single spirometric measures alone [43]. Biomarker or imaging integration represents another promising frontier; coupling lung age with genomic, proteomic, or radiomic data could uncover pathophysiologic pathways of accelerated lung aging or detect preclinical forms of lung disease [44]. Finally, as telehealth platforms and wearable respiratory sensors proliferate, there may be opportunities to develop simplified lung age offshoots—perhaps relying on robust but easily recorded flow-volume data—that can be administered remotely, expanding access to preventive care and early disease detection [45]. There is also potential in the longevity medicine field to create physiologic age scores with the help of models, such as lung age.

In conclusion, this study advances the notion that machine learning can transform large volumes of PFT data into a cohesive and clinically actionable index of respiratory health. The novelty of time-series analysis, combined with interpretability frameworks, promises not only more accurate estimations of lung age but also a deeper understanding of sex-related physiological differences. Over the long term, we postulate that lung age, particularly in its more advanced configurations, could emerge as a powerful surrogate measure for disease severity and prognosis, further refining how clinicians and patients perceive and manage respiratory health. As we continue to validate lung age and sex in broader populations and more diverse clinical contexts, the prospect of integrating machine learning–driven insights into routine pulmonary care draws closer to reality, potentially revolutionizing how we monitor and intervene in chronic lung conditions.

## Supplementary material

10.2196/89060 Multimedia Appendix 1 Supplemental material.

## Abbreviations

AUC: area under the curve
COPD: chronic obstructive pulmonary disease
FEV1: forced expiratory volume in 1 second
FVC: forced vital capacity
IPF: idiopathic pulmonary fibrosis
MAE: mean absolute error
PFT: pulmonary function test
RMSE: root mean square error
ROC: receiver operating characteristic curve
RV: residual volume
SHAP: Shapley additive explanations
TLC: total lung capacity
