# Supplementary material for: Estimating a Physiological Lung Function Score and Biological Sex Using Pulmonary Function Tests and Machine Learning: Retrospective Study
Source: JMIR AI. 2026 Jun 1;5:e89060. doi: 10.2196/89060 (PMC13224920; doi:10.2196/89060)

**Supplemental Information**

**Supplemental Table 1: Model features**

| **Model** | **Predictors** |
| --- | --- |
| Model 1 | (Age\|Sex)^*^, Weight, Height, Race, Forced Vital Capacity, Forced Expiratory Volume (1 Sec), FEV1/FVC Ratio |
| Model 2 | (Age\|Sex)^*^, Weight, Height, Race, Slow Vital Capacity, Forced Vital Capacity, Forced Expiratory Volume (1 Sec), FEV1/FVC Ratio, Peak Expiratory Flow, Forced Expiratory Time |
| Model 3 | (Age\|Sex)^*^, Weight, Height, Race, Slow Vital Capacity, Forced Vital Capacity, Forced Expiratory Volume (1 Sec), FEV1/FVC Ratio, Peak Expiratory Flow, Forced Expiratory Time, Functional Residual Capacity, Residual Volume, Total Lung Capacity, RV as % of TLC, Vital Capacity, Diffusing Capacity, Alveolar Volume, Corrected Diffusing Capacity |
| Model 4 | (Age\|Sex)^*^, Weight, Height, Race, Slow Vital Capacity, Forced Vital Capacity, Forced Expiratory Volume (1 Sec), FEV1/FVC Ratio, Peak Expiratory Flow, Forced Expiratory Time, Functional Residual Capacity, Residual Volume, Total Lung Capacity, RV as % of TLC, Vital Capacity, Diffusing Capacity, Alveolar Volume, Corrected Diffusing Capacity, Lag-1 Autocorrelation, Average Autocorrelation (Lags 1-10), Lag-1 Autocorrelation (1st Difference), Average Autocorrelation (1st Difference, Lags 1-10), Lag-1 Autocorrelation (2nd Difference), Average Autocorrelation (2nd Difference, Lags 1-10), Spectral Entropy, Strength of Trend, Spikiness, Linearity, Curvature, Lag-1 Autocorrelation (Residuals), Average Autocorrelation (Residuals, Lags 1-10), ARCH LM Statistic, Mode of Histogram (10 Bins), Outlier Proportion (MD-RMD), Hurst Exponent, Stability (Variance Changes Over Time), Lumpiness (Variance Between Segments), Largest Level Shift, Time of Largest Level Shift, Largest Variance Shift, Time of Largest Variance Shift, Largest Kullback-Leibler Divergence Shift, Time of Largest KL Divergence Shift, Nonlinearity Score, KPSS Unit Root Test Statistic, Phillips-Perron Unit Root Test Statistic, Proportion of Zeros |
| ^*^For the Age model, Sex was included as a predictor. For the Sex model, Age was included as a predictor. | |

**Supplemental Table 2: Lung age performance metrics**

| Model | RMSE | MAE | R2 |
| --- | --- | --- | --- |
| Model 1 | 9.29 (8.87-9.68) | 7.32 (6.99-7.65) | 0.65 (0.62-0.68) |
| Model 2 | 9.13 (8.74-9.49) | 7.27 (6.96-7.58) | 0.67 (0.63-0.70) |
| Model 3 | 7.19 (6.90-7.49) | 5.69 (5.45-5.95) | 0.79 (0.77-0.81) |
| Model 4 | 7.01 (6.73-7.30) | 5.55 (5.32-5.80) | 0.80 (0.78-0.82) |
| Parkes et al. | 13.87 (13.34-14.36) | 11.08 (10.62-11.54) | 0.23 (0.15-0.31) |

**Supplemental Table 3:** **Predicted lung age percentiles by chronological age for Model 3 and Model 1.**

|  | Model 3 | | | Model 1 | | |
| --- | --- | --- | --- | --- | --- | --- |
| Chronological Age (yrs) | 10th Percentile | 50th Percentile (Median) | 90th Percentile | 10th Percentile | 50th Percentile (Median) | 90th Percentile |
| 20 | 18.3 | 23.1 | 30.6 | 21.6 | 29.6 | 43.3 |
| 30 | 27.4 | 32.7 | 40.0 | 28.3 | 37.1 | 48.9 |
| 40 | 36.4 | 42.0 | 49.0 | 35.3 | 44.5 | 54.7 |
| 50 | 45.2 | 51.1 | 57.5 | 42.4 | 52.0 | 60.8 |
| 60 | 54.0 | 60.0 | 65.7 | 49.9 | 59.5 | 67.2 |
| 70 | 62.7 | 68.6 | 73.5 | 57.5 | 67.0 | 73.8 |
| 80 | 71.3 | 77.0 | 80.9 | 65.4 | 74.5 | 80.7 |
| 90 | 79.8 | 85.2 | 87.8 | 73.5 | 82.0 | 87.9 |

**Supplemental Table 4: Sex model performance with 95% confidence intervals**

| Model | AUC | Sensitivity | Specificity | PPV | NPV |
| --- | --- | --- | --- | --- | --- |
| Model 1 | 0.965 (0.955, 0.975) | 88.3% (85.2%, 90.9%) | 93.4% (91.4%, 95.1%) | 90.7% (87.8%, 93.0%) | 91.7% (89.4%, 93.6%) |
| Model 2 | 0.978 (0.970, 0.986) | 90.5% (87.7%, 92.9%) | 94.8% (92.9%, 96.3%) | 92.6% (90.0%, 94.7%) | 93.3% (91.2%, 95.0%) |
| Model 3 | 0.981 (0.974, 0.988) | 91.1% (88.3%, 93.4%) | 95.1% (93.2%, 96.5%) | 93.0% (90.5%, 95.1%) | 93.7% (91.7%, 95.3%) |
| Model 4 | 0.981 (0.975, 0.988) | 91.7% (89.0%, 93.9%) | 95.6% (93.9%, 97.0%) | 93.8% (91.4%, 95.7%) | 94.1% (92.1%, 95.7%) |

**Supplemental Table 5: Age-stratified performance for Model 4**

| Age (years) | n | RMSE | MAE | Mean signed error | Median signed error |
| --- | --- | --- | --- | --- | --- |
| 18-29 | 92 | 8.73 (7.42 to 9.88) | 6.90 (5.82 to 7.98) | 4.64 (3.08 to 6.08) | 4.48 (2.62 to 6.54) |
| 30-39 | 103 | 9.07 (7.96 to 10.25) | 7.38 (6.41 to 8.42) | 4.34 (2.87 to 5.80) | 4.51 (2.42 to 6.74) |
| 40-49 | 185 | 7.24 (6.51 to 7.95) | 5.77 (5.14 to 6.42) | 2.40 (1.44 to 3.39) | 2.55 (1.48 to 3.95) |
| 50-59 | 289 | 6.42 (5.82 to 6.99) | 5.07 (4.60 to 5.53) | 1.06 (0.33 to 1.77) | 1.31 (0.28 to 2.34) |
| 60-69 | 330 | 5.69 (5.30 to 6.11) | 4.60 (4.26 to 4.96) | -1.00 (-1.62 to -0.39) | -1.15 (-1.97 to -0.36) |
| 70-79 | 204 | 7.15 (6.48 to 7.81) | 5.69 (5.10 to 6.30) | -3.92 (-4.73 to -3.13) | -3.49 (-5.09 to -2.15) |
| 80+ | 55 | 8.06 (6.93 to 9.22) | 6.81 (5.74 to 7.97) | -6.47 (-7.78 to -5.22) | -6.14 (-7.97 to -4.84) |

**Supplemental Table 6: Age-stratified performance for Model 3**

| Age (years) | n | RMSE | MAE | Mean signed error | Median signed error |
| --- | --- | --- | --- | --- | --- |
| 18-29 | 92 | 9.35 (8.04 to 10.56) | 7.60 (6.53 to 8.70) | 5.80 (4.29 to 7.29) | 5.87 (4.19 to 7.61) |
| 30-39 | 103 | 9.23 (8.16 to 10.30) | 7.56 (6.57 to 8.58) | 4.14 (2.65 to 5.70) | 3.98 (1.70 to 6.74) |
| 40-49 | 185 | 7.41 (6.71 to 8.09) | 5.94 (5.31 to 6.60) | 2.42 (1.43 to 3.45) | 2.42 (1.25 to 3.68) |
| 50-59 | 289 | 6.49 (5.89 to 7.06) | 5.15 (4.69 to 5.60) | 1.12 (0.37 to 1.85) | 1.49 (0.67 to 2.46) |
| 60-69 | 330 | 5.74 (5.33 to 6.16) | 4.63 (4.27 to 5.00) | -1.13 (-1.76 to -0.52) | -1.10 (-2.07 to -0.47) |
| 70-79 | 204 | 7.39 (6.65 to 8.11) | 5.80 (5.17 to 6.44) | -4.03 (-4.89 to -3.19) | -3.58 (-4.99 to -2.56) |
| 80+ | 55 | 8.48 (7.21 to 9.73) | 6.98 (5.79 to 8.29) | -6.72 (-8.13 to -5.43) | -5.83 (-8.11 to -4.59) |

**Supplemental Table 7: Age model anthropometric ablation**

| Model | Variant | RMSE | MAE | R2 |
| --- | --- | --- | --- | --- |
| Model 3 | Original | 7.19 (6.90-7.49) | 5.69 (5.45-5.95) | 0.79 (0.77-0.81) |
| Model 3 | No anthropometry | 7.36 (7.06-7.68) | 5.79 (5.55-6.05) | 0.78 (0.76-0.80) |
| Model 4 | Original | 7.01 (6.73-7.30) | 5.55 (5.32-5.80) | 0.80 (0.78-0.82) |
| Model 4 | No anthropometry | 7.10 (6.82-7.42) | 5.59 (5.35-5.85) | 0.80 (0.78-0.82) |

**Supplemental Table 8: Sex model anthropometric ablation**

| Model | Variant | AUC | Sensitivity | Specificity | PPV | NPV |
| --- | --- | --- | --- | --- | --- | --- |
| Model 3 | Original | 0.981 (0.974, 0.988) | 91.1% (88.3%, 93.4%) | 95.1% (93.2%, 96.5%) | 93.0% (90.5%, 95.1%) | 93.7% (91.7%, 95.3%) |
| Model 3 | No anthropometry | 0.980 (0.973, 0.986) | 91.5% (88.8%, 93.7%) | 95.1% (93.2%, 96.5%) | 93.1% (90.5%, 95.1%) | 93.9% (91.9%, 95.5%) |
| Model 4 | Original | 0.981 (0.975, 0.988) | 91.7% (89.0%, 93.9%) | 95.6% (93.9%, 97.0%) | 93.8% (91.4%, 95.7%) | 94.1% (92.1%, 95.7%) |
| Model 4 | No anthropometry | 0.981 (0.974, 0.987) | 91.5% (88.8%, 93.7%) | 95.2% (93.4%, 96.6%) | 93.2% (90.7%, 95.2%) | 93.9% (91.9%, 95.5%) |

**Supplemental Table 9: Final tuned hyperparameters**

| Outcome | Model | Learn rate | Max depth | Row sample rate | Col sample rate | Trees | CV metric |
| --- | --- | --- | --- | --- | --- | --- | --- |
| Age | Model 1 | 0.01 | 5 | 0.80 | 0.85 | 1,000 | RMSE = 9.123 |
| Age | Model 2 | 0.01 | 5 | 0.80 | 0.85 | 1,000 | RMSE = 8.904 |
| Age | Model 3 | 0.01 | 5 | 0.80 | 0.85 | 2,500 | RMSE = 7.161 |
| Age | Model 4 | 0.01 | 5 | 0.80 | 0.85 | 2,500 | RMSE = 7.068 |
| Sex | Model 1 | 0.01 | 5 | 0.80 | 0.85 | 1,000 | AUC = 0.9734 |
| Sex | Model 2 | 0.01 | 5 | 0.80 | 0.85 | 1,000 | AUC = 0.9809 |
| Sex | Model 3 | 0.01 | 5 | 0.80 | 0.85 | 1,000 | AUC = 0.9828 |
| Sex | Model 4 | 0.01 | 5 | 0.80 | 0.85 | 2,500 | AUC = 0.9829 |

**Supplemental Table 10: Sex model calibration metrics**

| Model | Brier score | ECE | Spiegelhalter z | P-value |
| --- | --- | --- | --- | --- |
| Model 1 | 0.066 (0.056-0.077) | 0.020 (0.016-0.039) | 3.06 | **0.002** |
| Model 2 | 0.052 (0.043-0.062) | 0.018 (0.014-0.034) | 2.83 | **0.005** |
| Model 3 | 0.050 (0.041-0.059) | 0.018 (0.013-0.033) | 2.85 | **0.004** |
| Model 4 | 0.049 (0.039-0.060) | 0.031 (0.024-0.046) | 8.92 | **<0.001** |

**Supplemental Figure 1. ROC curves for sex prediction using Models 3 and 1.**

Receiver operating characteristic (ROC) curves are shown for the discrete PFT model (Model 3, left) and the basic spirometry model (Model 1, right). Model 3 achieved an AUC of 0.981 (95% CI: 0.974–0.988), while Model 1 achieved an AUC of 0.965 (95% CI: 0.955–0.975). Annotated metrics include classification accuracy, sensitivity, specificity, positive predictive value (PPV), and negative predictive value (NPV).

**
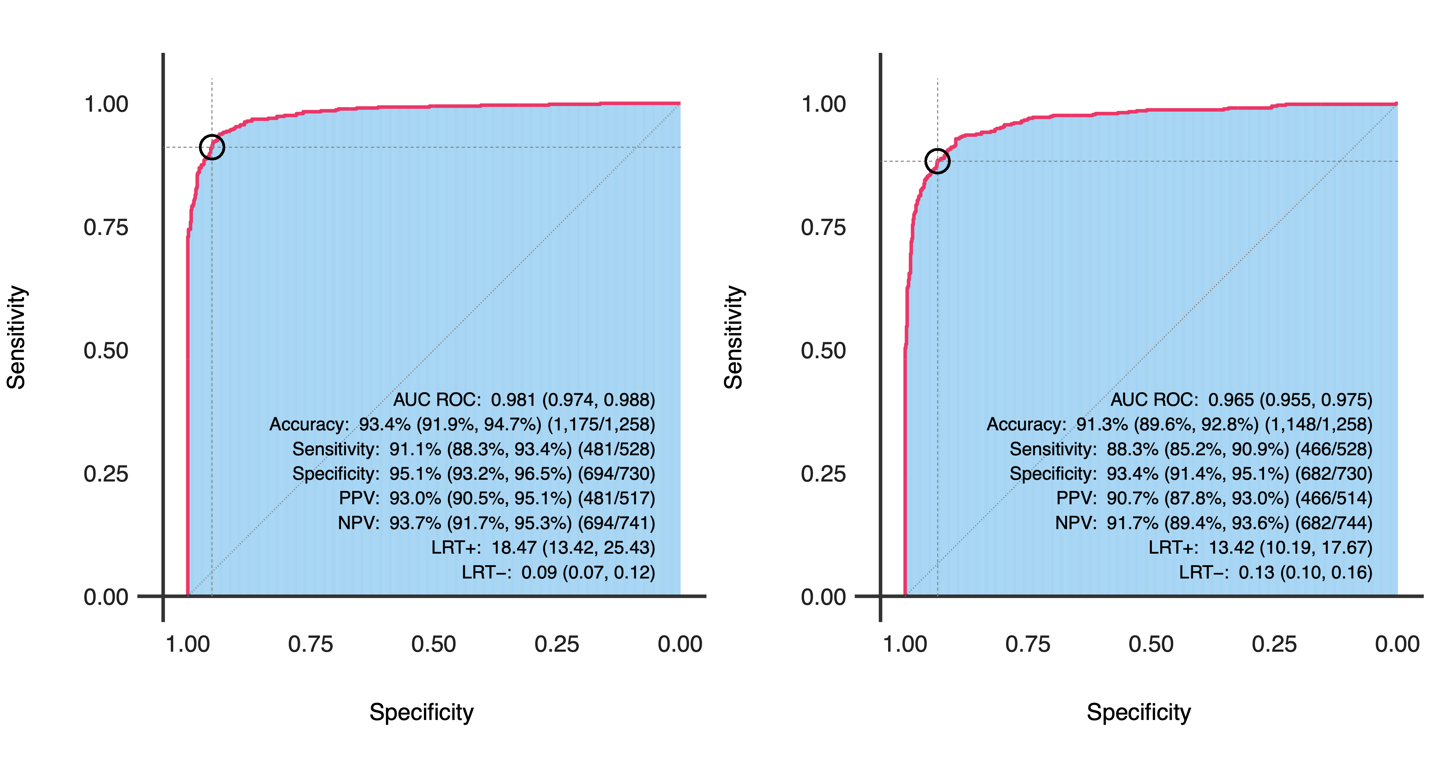
**

**Supplemental Figure 2: Signed prediction error by age group for Model 4.**

Boxplots show signed prediction error for the time-series-feature age model (Model 4) within each 10-year age group on the independent test set. Signed error was defined as predicted age minus chronological age. The horizontal dashed line indicates zero error; positive values indicate overestimation of lung age and negative values indicate underestimation.


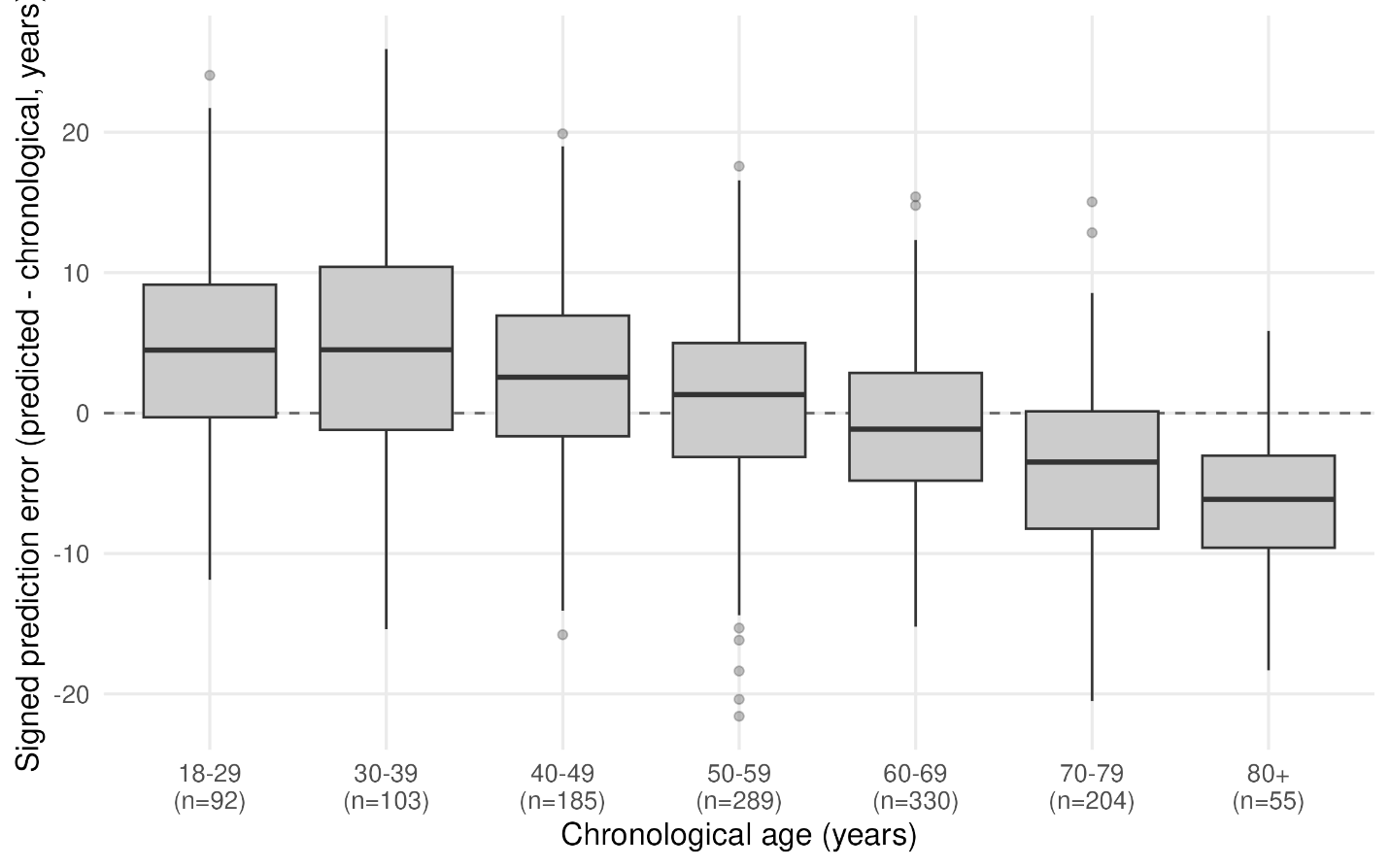


**Supplemental Figure 3: Signed prediction error versus chronological age for Model 4.**

Each point represents one patient in the independent test set for the time-series-feature age model (Model 4). Signed error was defined as predicted age minus chronological age. The solid curve shows a loess smoother with a 95% confidence interval ribbon. Positive values indicate overestimation of lung age and negative values indicate underestimation.


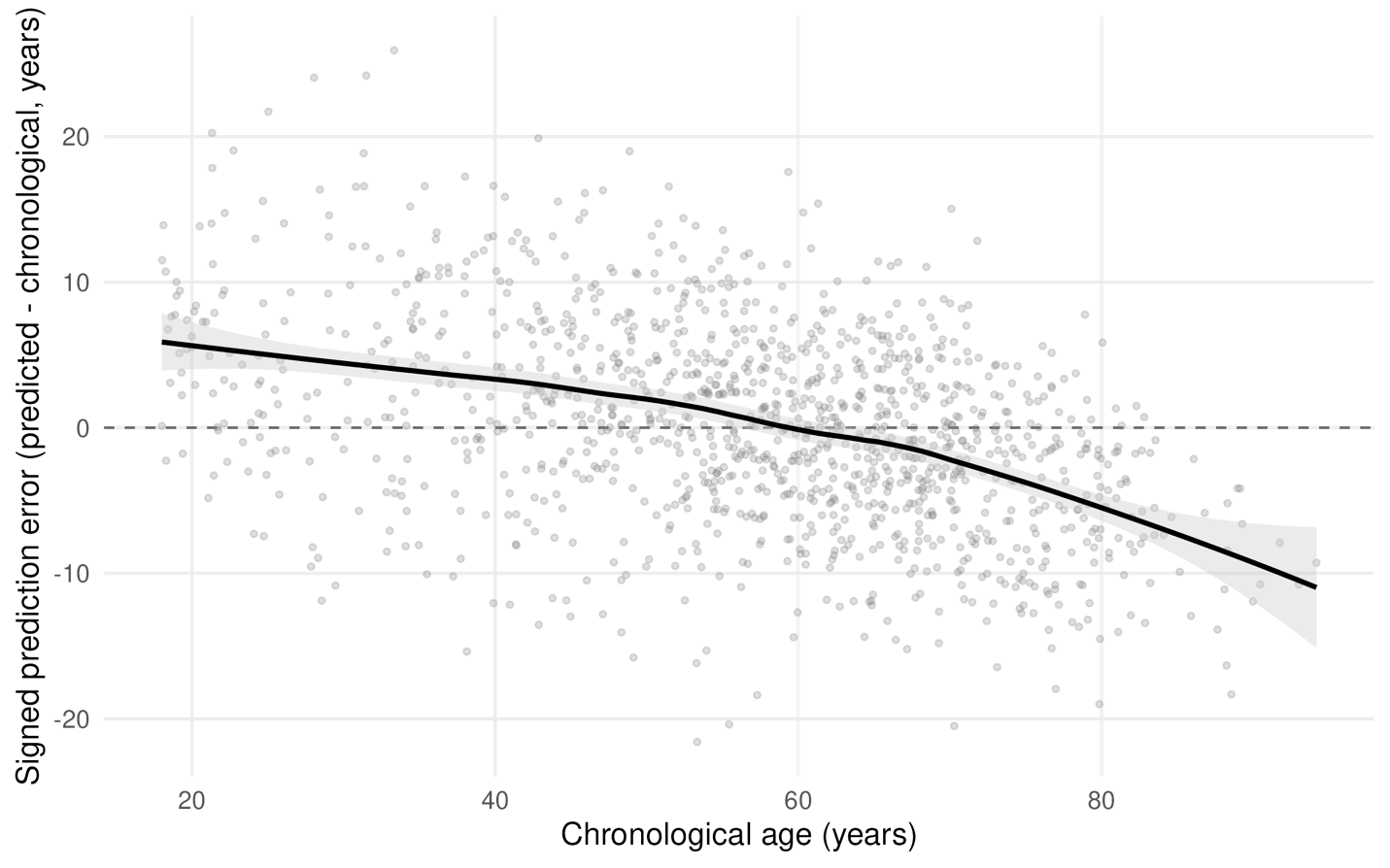


**Supplemental Figure 4: Signed prediction error by age group for Model 3.**

Boxplots show signed prediction error for the all-tabular-feature age model (Model 3) within each 10-year age group on the independent test set. Signed error was defined as predicted age minus chronological age. The horizontal dashed line indicates zero error; positive values indicate overestimation of lung age and negative values indicate underestimation.

**
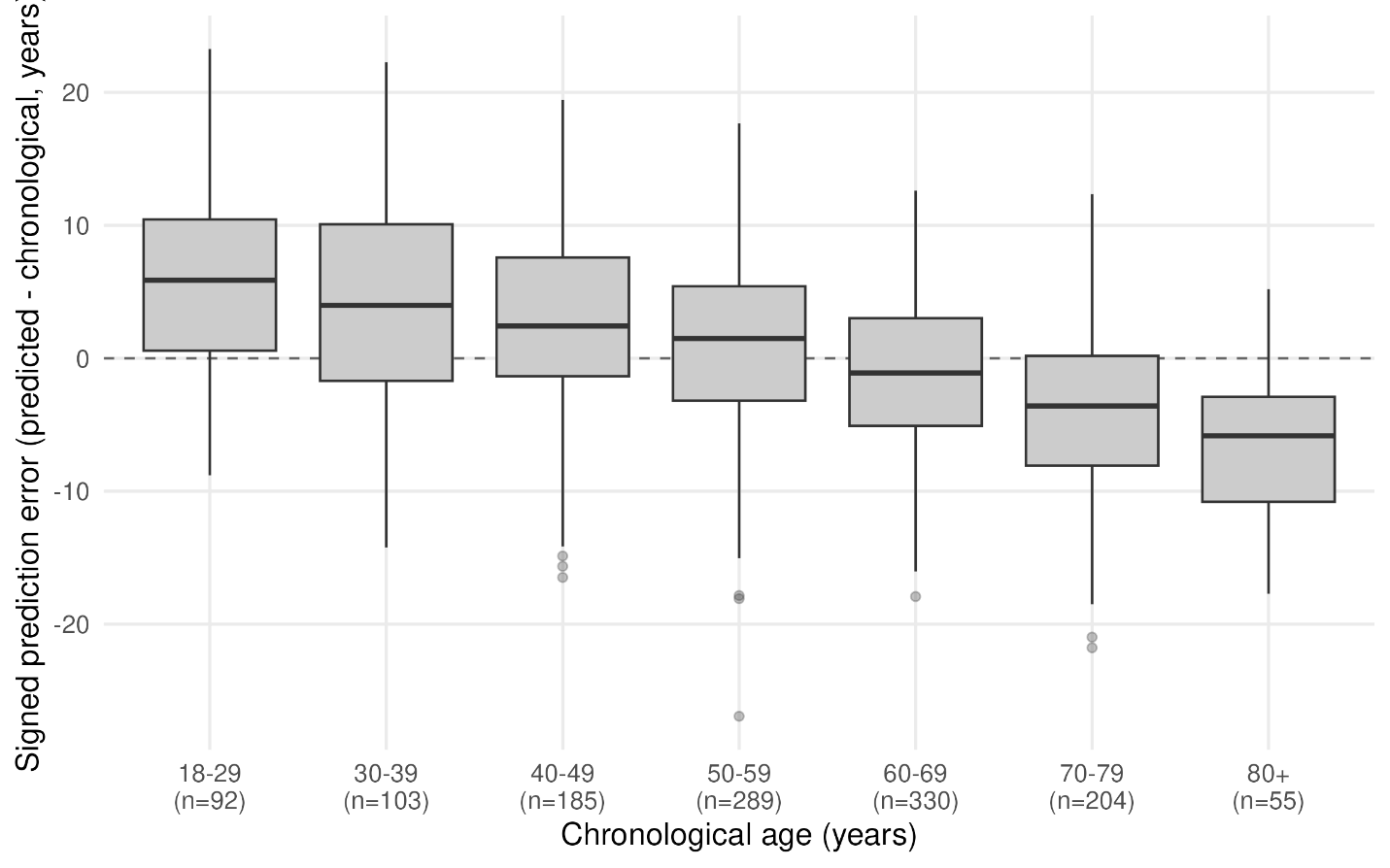
**

**Supplemental Figure 5: Signed prediction error versus chronological age for Model 3.**

Each point represents one patient in the independent test set for the all-tabular-feature age model (Model 3). Signed error was defined as predicted age minus chronological age. The solid curve shows a loess smoother with a 95% confidence interval ribbon. Positive values indicate overestimation of lung age and negative values indicate underestimation.

**
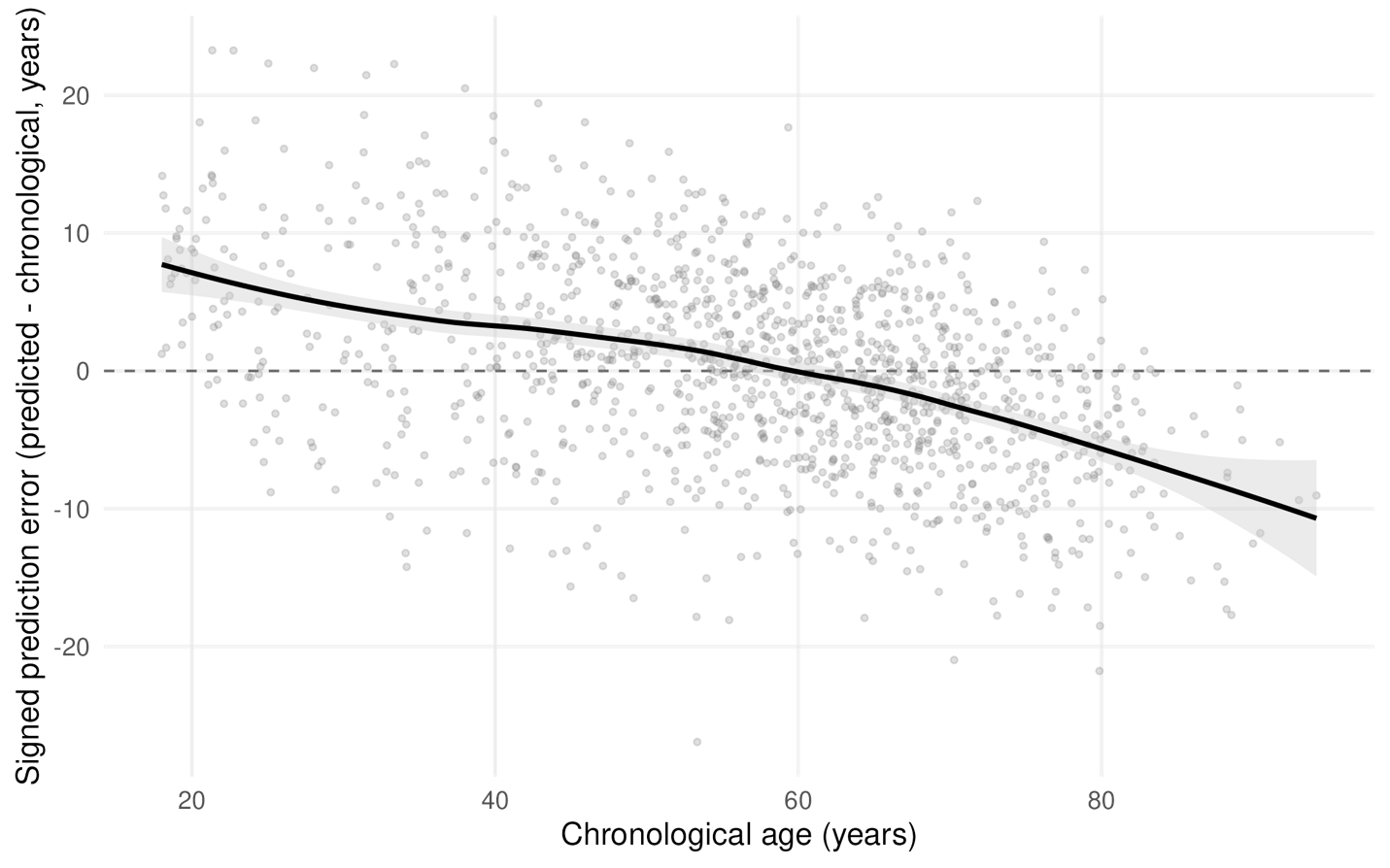
**

**Supplemental Figure 6: Reliability diagrams for sex classification models.**

Reliability diagrams are shown for each of the four sex classification models on the independent test set. Predicted probabilities of male sex were grouped into 10 equal-width probability bins. Each point shows the mean predicted probability within a bin plotted against the observed proportion of male patients in that bin. Error bars indicate 95% confidence intervals for the observed proportion, and the dashed line indicates perfect calibration.


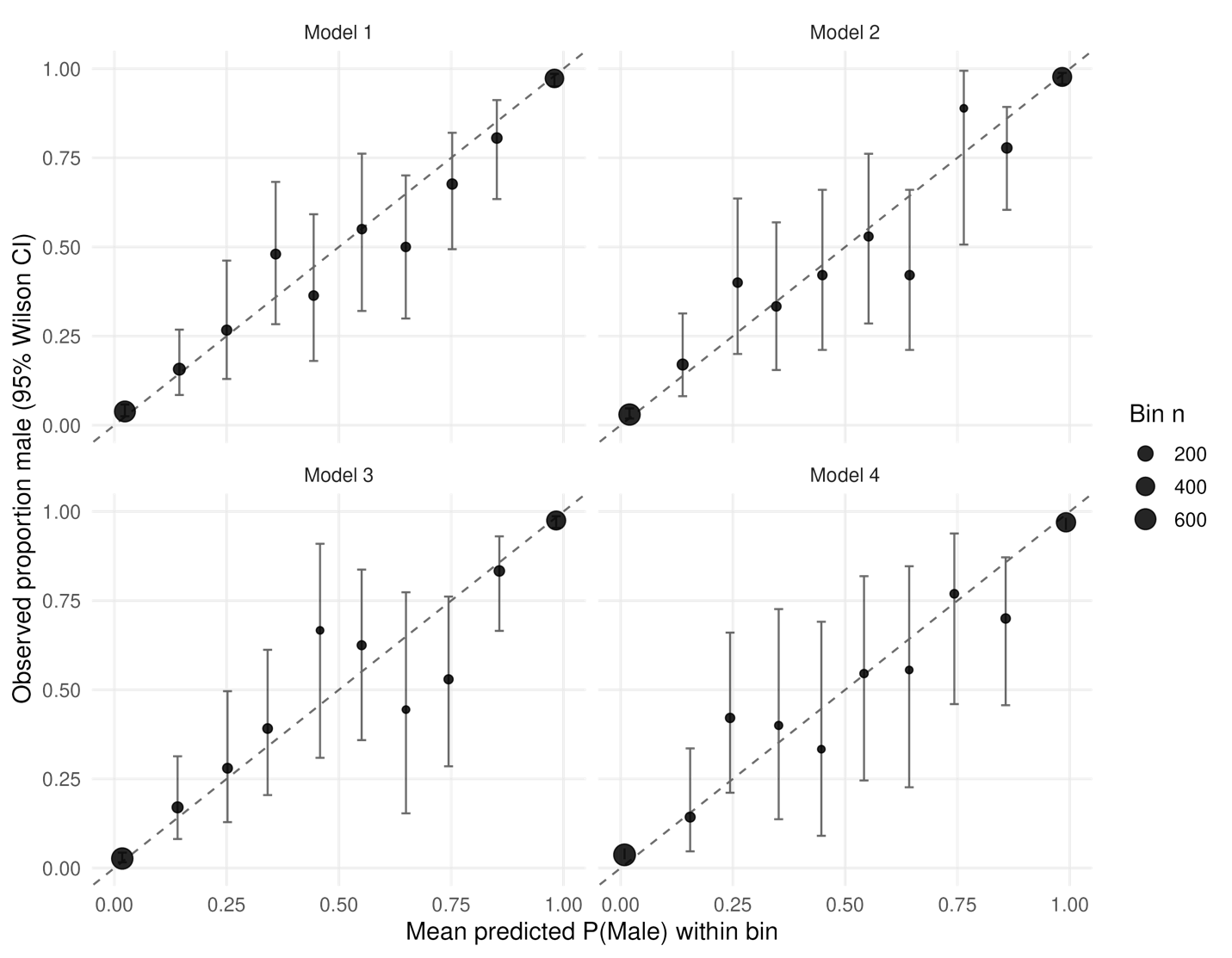

Supplement: Multimedia Appendix 1 [file ai-v5-e89060-s001.docx]
